# Supplementary material for: Infants’ cortex undergoes microstructural growth coupled with myelination during development
Source: Commun Biol. 2021 Oct 14;4:1191. doi: 10.1038/s42003-021-02706-w (PMC8516989; doi:10.1038/s42003-021-02706-w)
Supplement: Supplementary file 2 — Supplementary Information [file 42003_2021_2706_MOESM2_ESM.pdf]

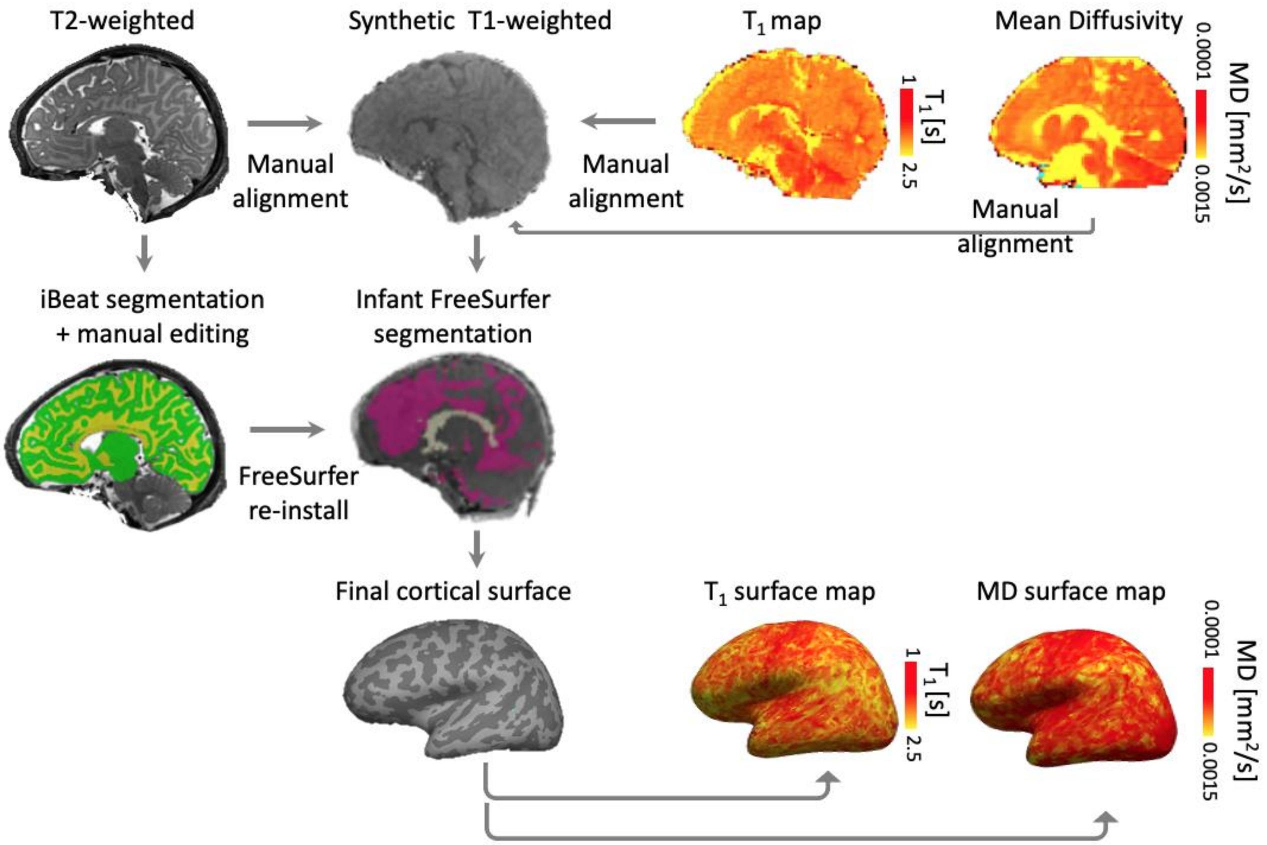

Supplementary Figure 1. MRI data preprocessing pipeline. Schematic showing the preprocessing pipeline associated with (i) obtaining the white and gray matter segmentations for generating the cortical surfaces, and (ii) generating quantitative T_1 and mean diffusivity (MD) maps aligned to the same anatomical brain volume and cortical surface. All analyses are done for each individual baby and timepoint in their individual brain space.

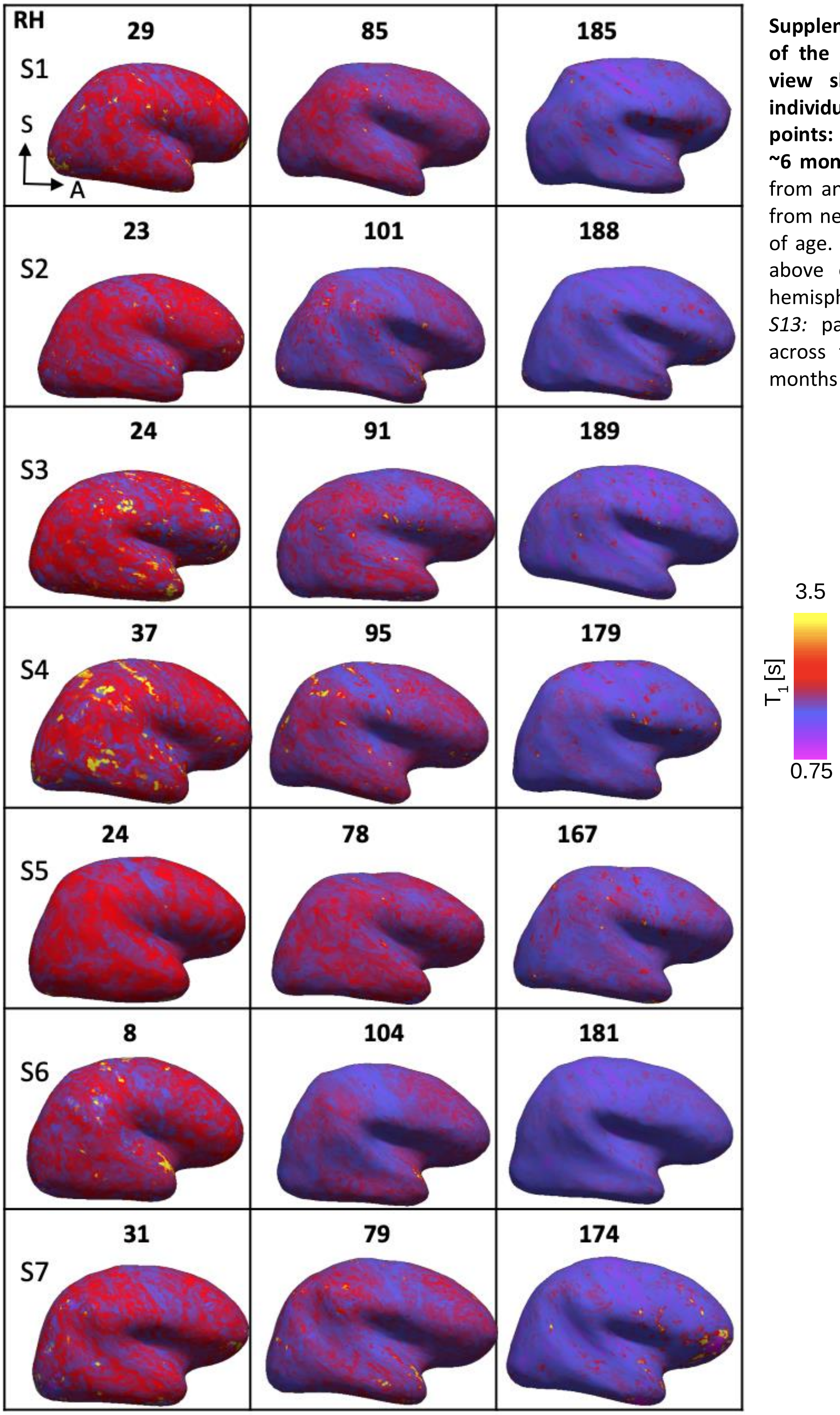

Supplementary Figure 2. Cortical surfaces of the right hemisphere in the sagittal view showing T_1 development in all individual babies across three time points: newborn, ~3 months of age, and ~6 months of age. Each row shows data from an individual baby. T_1 [s] decreases from newborn (red) to 6 months (purple) of age. Age at scan (in days) is indicated above each cortical surface. *RH:* right hemisphere*, S:* superior, *A:* anterior. *S1-S13:* participant number (*N*=13 infants across three time points: newborn, ~3 months of age, and ~6 months of age.

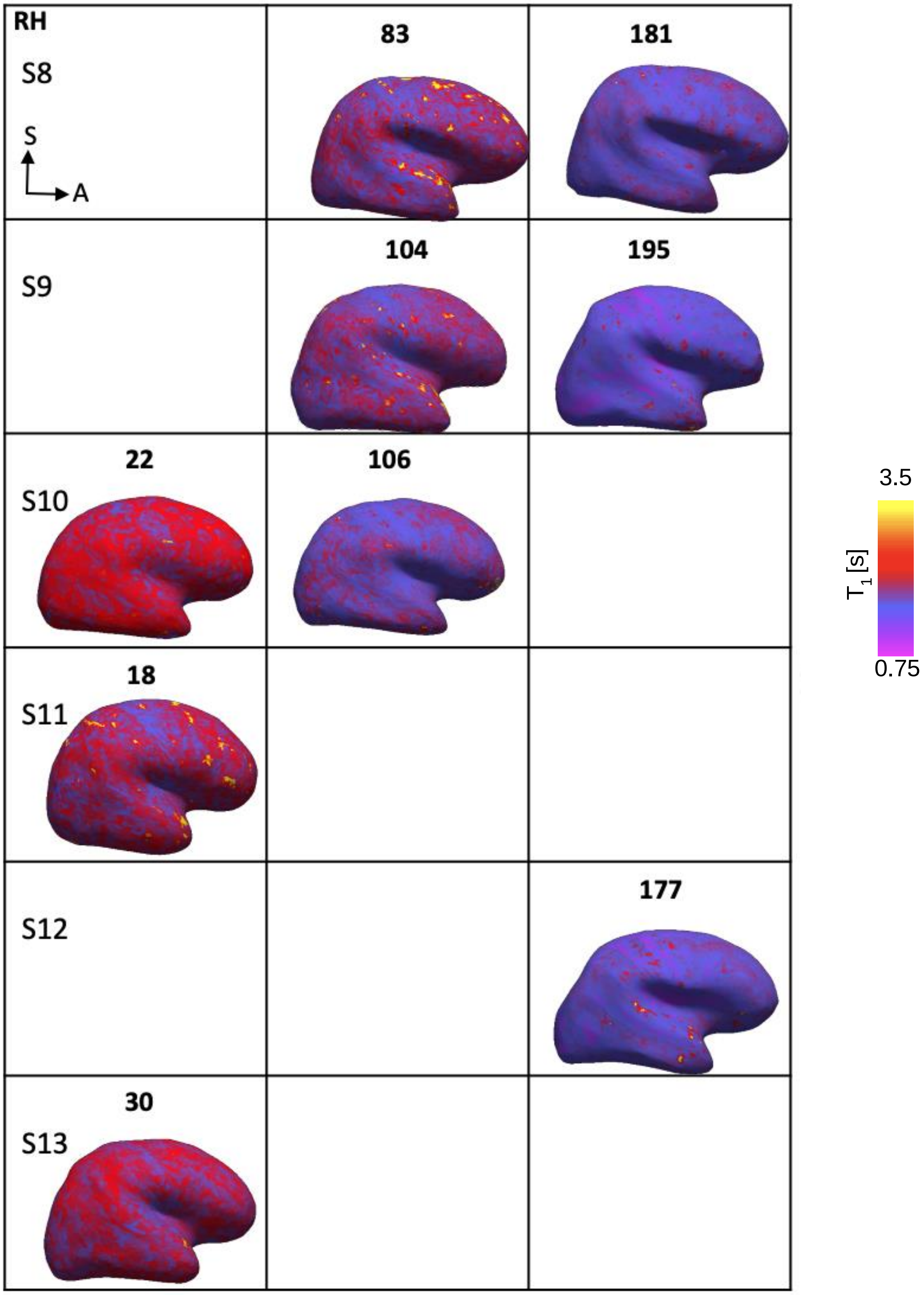
RH
S8
S
A
83
181
S9
104
195
S10
22
106
S11
18
S12
177
S13
30
3.5
T1 [s]
0.75

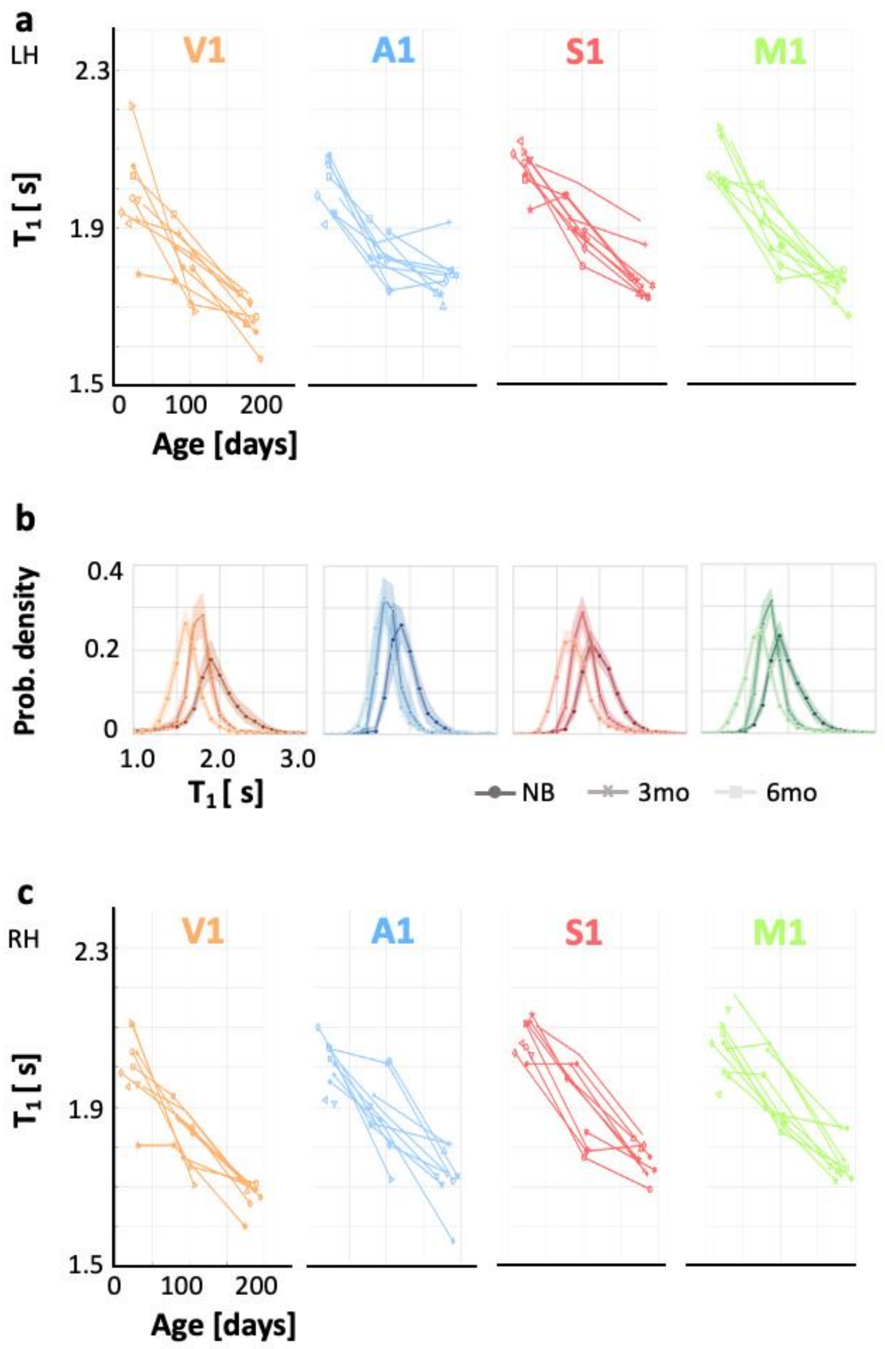

Supplementary Figure 3. Mean T_1 in four primary sensory regions decreases with age in the first six months of infant life. (a) Significant decrease in T_1 with age are observed in the four primary sensory-motor cortices of the left hemisphere. *V1:* primary visual cortex*, A1:* primary auditory cortex, *S1:* primary somatosensory cortex, *M1:* primary motor cortex); slopes and significance (p-values) in **Supplementary Table 2**. Each line connects the measurements across time in an individual infant (participant is indicated by symbol). (b) Distribution of T_1 across voxels in each area and timepoint in the left hemisphere. Distributions are calculated within participant, and then averaged across participants. Data show that in primary sensory-motor cortices T_1 of individual infants decreases from newborns (darker colors) to 6 months of age (lighter colors). *Solid lines:* mean distribution across participants; *shaded region:* standard error of the mean (SE) across 10 infants in each timepoint. *NB:* newborn; *3 mo:* ~3-month-old; *6 mo:* ~6-month-old. (c) Same as (a) for right hemisphere data. For panels a,c, N_{total} = 30, 10 infants at each time point (newborn, ~3 months, ~6 months). *LH:* left hemisphere. *RH:* right hemisphere.

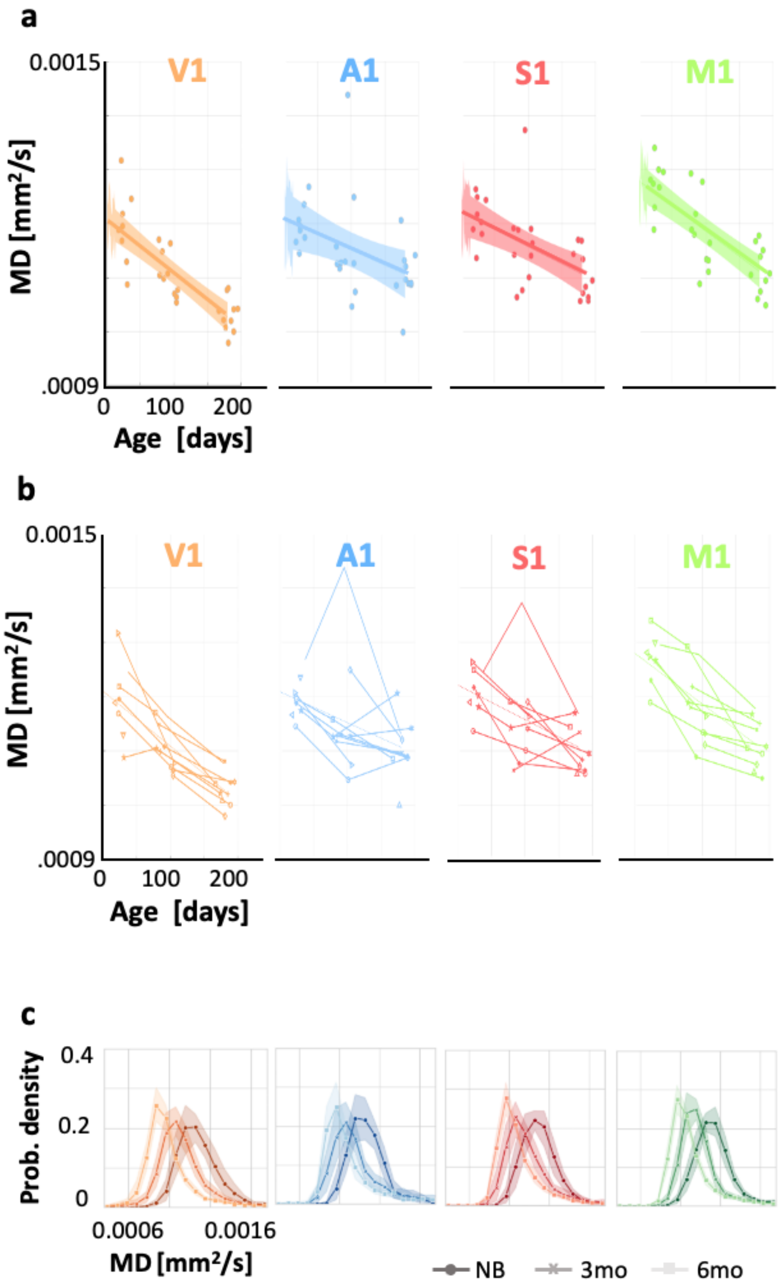

Supplementary Figure 4. Mean diffusivity (MD) in four primary sensory regions decreases with age in the first six months of infant life. All data shown here are from the left hemisphere**.** (a) Significant decrease in MD with age in the four primary sensory-motor cortices. *V1:* primary visual area, *A1:* primary auditory area, *S1:* primary somatosensory area*, M1:* primary motor area. Slopes and p-values in **Supplementary Table 3**. Each dot represents the mean MD of an infant's region of interest. Lines represent linear mixed model (LMM) fits. Shaded portions represent 95% confidence intervals of the LMMs. (b) Similar to (a) with lines connecting data of individual infant (indicated by symbol) across time points. For figs. a,b, N_{total} = 30, 10 infants at each time point (newborn, ~3 months, ~6 months). (c) Distribution of MD across voxels in each primary sensory-motor area and timepoint. In all cortices and both hemispheres, mean MD significantly decreases from 0.0012±6.29x10^{-5} [mm^2/s] (mean $\pm$ standard deviation) at birth to 0.0011±7.51x10^{-5} [mm^2/s] at 3 months to 0.0010±6.5x10^{-5} [mm^2/s] at 6 months. *Solid lines:* indicate mean, *shaded region:* indicates standard error across 10 participants at each timepoint. *NB:* newborn; *3 mo:* ~3-month-old; *6 mo:* ~6-month-old.

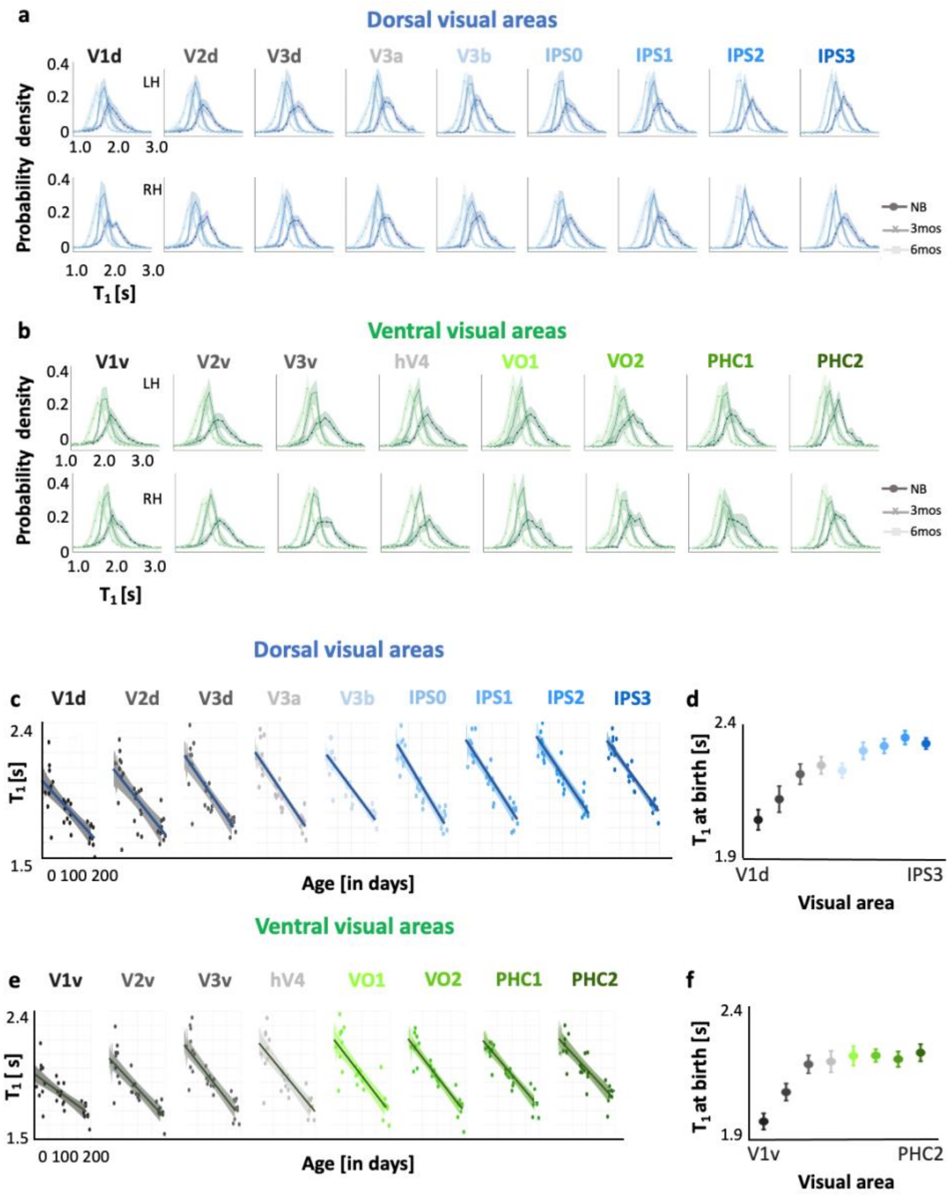

Supplementary Figure 5. Hierarchical T_1 development in the dorsal and ventral visual streams during early infancy. (a,b) Distributions of T_1 in dorsal (a) and ventral (b) visual areas in the left (LH) and right (RH) hemispheres shift leftward with age in the first six months of infant life. Solid lines indicate mean, shaded region indicates standard error across 10 participants at each timepoint *NB:* newborn; *3 mos:* ~3-month-old; *6 mos:* ~6-month-old. (c,e) Developmental trajectory of T_1 in the first 6 months of life. (c-f) Left hemisphere data. (c) Dorsal stream (V1d to IPS3). (e) Ventral stream (V1v to PHC2). Each dot represents mean T_1 per region of interest (ROI) per infant. For figs. c,e, N_{total} = 30, 10 infants at each time point (newborn, ~3 months, ~6 months). Lines represent LMM fits. Shaded portions represent 95% confidence intervals from the LMM. As in the right hemisphere, T_1 in the left hemisphere linearly decreases in all ventral and dorsal visual areas. (d,f) Estimated mean T_1 of each area at birth (intercepts of LMMs per ROI) in the dorsal (d) and ventral (f) streams in the left hemisphere. Data reveal a hierarchical development, with lower T_1 in earlier than later visual areas of these streams. *Error bars:* standard error on estimates of intercepts. Slopes and p-values in **Supplementary Table 4**. Comparison of LMM estimates of T_1 at birth across visual areas reveals that in both streams and hemispheres there is a gradual increase of cortical T_1 at birth across the visual hierarchy.

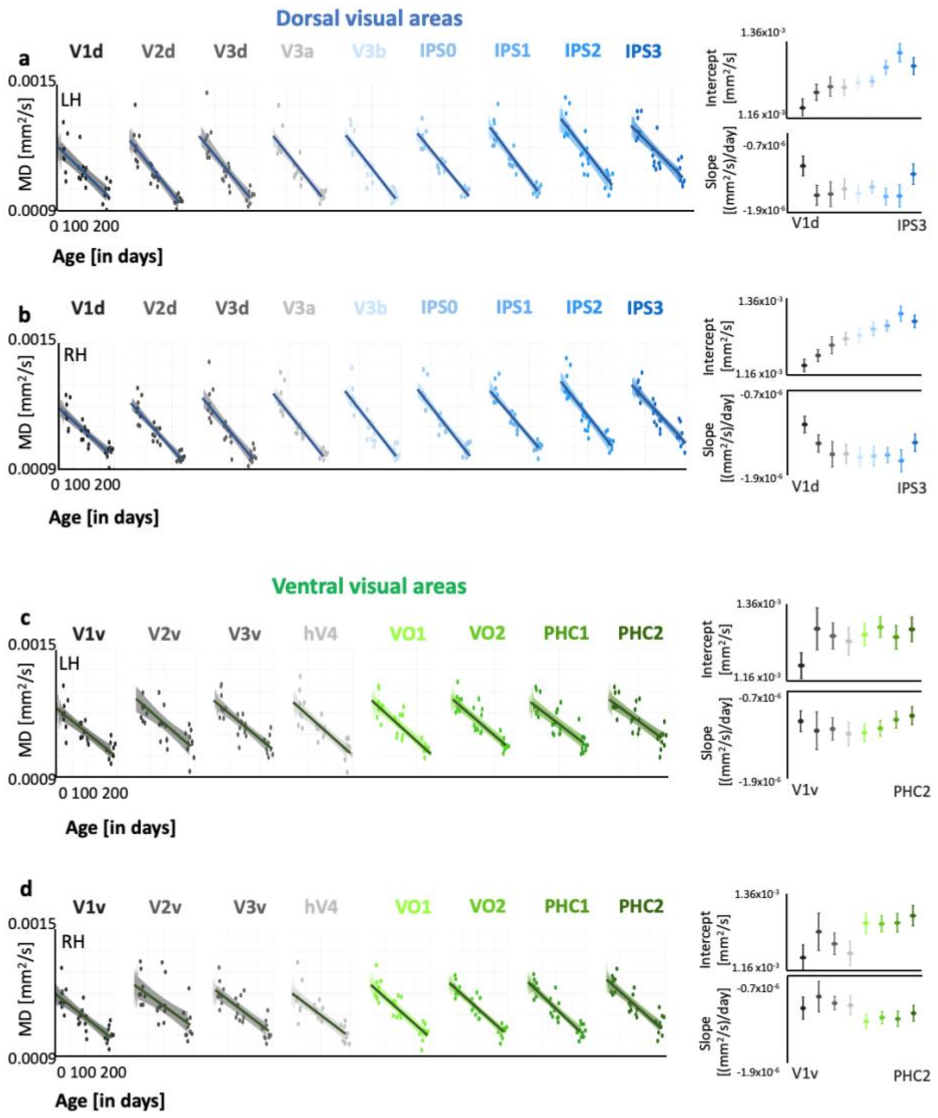

Supplementary Figure 6. Hierarchical development of mean diffusivity (MD) in the dorsal and ventral visual streams during early infancy. (a,b) Left to right shows the developmental trajectory of MD in the first 6 months of life, in the left (a) and right (b) dorsal visual streams (V1d to IPS3), respectively. MD significantly decreases in all dorsal visual areas; slopes and p-values are presented in **Supplementary Table 5**. Each dot represents mean MD per ROI per infant. Lines represent the LMM fit. Shaded portions represent 95% confidence intervals. The slopes and intercepts of each LMM are shown on the right-side of each panel. *Error bars:* standard error on estimates of intercepts and slopes. Mean MD at birth in dorsal (intercepts of LMM per ROI) shows a hierarchical development with lower MD in earlier than later areas of the processing stream. (c,d) same as (a,b) for ventral stream visual areas (V1v to PHC2). For panels a-d, N_{total} = 30, 10 infants at each time point (newborn, ~3 months, ~6 months). Comparison of estimates of MD at birth (LMM intercept) across visual areas shows a gradual increase of cortical MD at birth across the visual hierarchy in both the dorsal visual stream, from V1d left: $0.0012 \pm 2.2\times10^{-5}$ [mm^2/s], right $0.0012 \pm 1.6\times10^{-5}$ [mm^2/s] to IPS3 left: $0.0013 \pm 2.2\times10^{-5}$ [mm^2/s]; right: $0.0013 \pm 1.7\times10^{-5}$ [mm^2/s] and the ventral visual stream, from V1v left: $0.0012 \pm 1.8\times10^{-5}$ [mm^2/s], right:$0.0012 \pm 2.0\times10^{-5}$ [mm^2/s] to PHC2 left: $0.0013 \pm 1.7\times10^{-5}$ [mm^2/s]; right: $0.0013 \pm 1.6\times10^{-5}$[mm^2/s]. *LH*: left hemisphere. *RH:* right hemisphere.

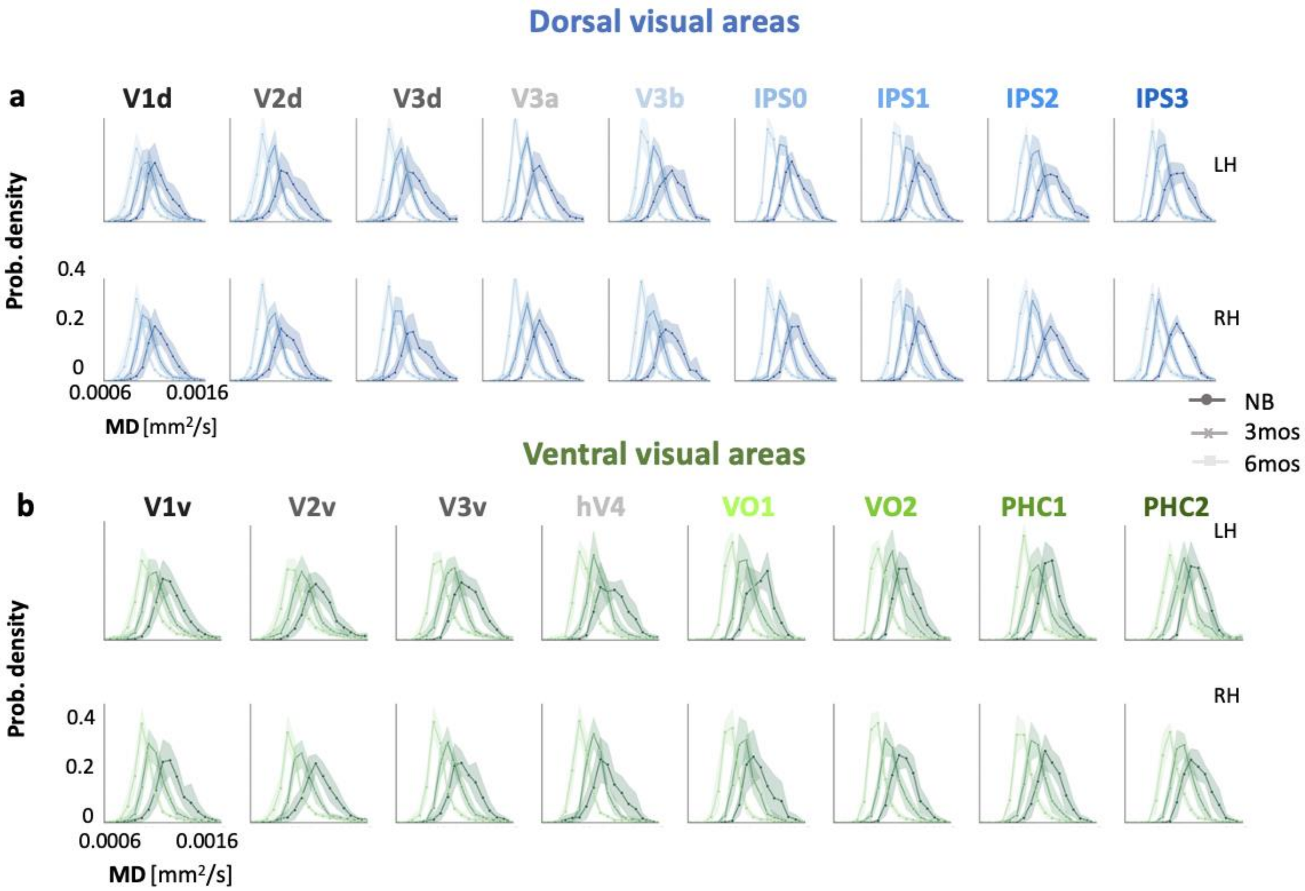

Supplementary Figure 7. Distributions of MD in dorsal and ventral visual regions in newborn (NB), 3-month-old (3mos) and 6-month-old (6mos) infants. (a,b) Distributions shift leftward with age in the first six months of infant life in both left and right hemispheres of dorsal (a) and ventral (b) stream visual areas, indicating the MD systematically decreases. Solid *lines:* mean across participants; *Shaded areas: standard error* of the mean across 10 participants. *LH*: left hemisphere. *RH:* right hemisphere.

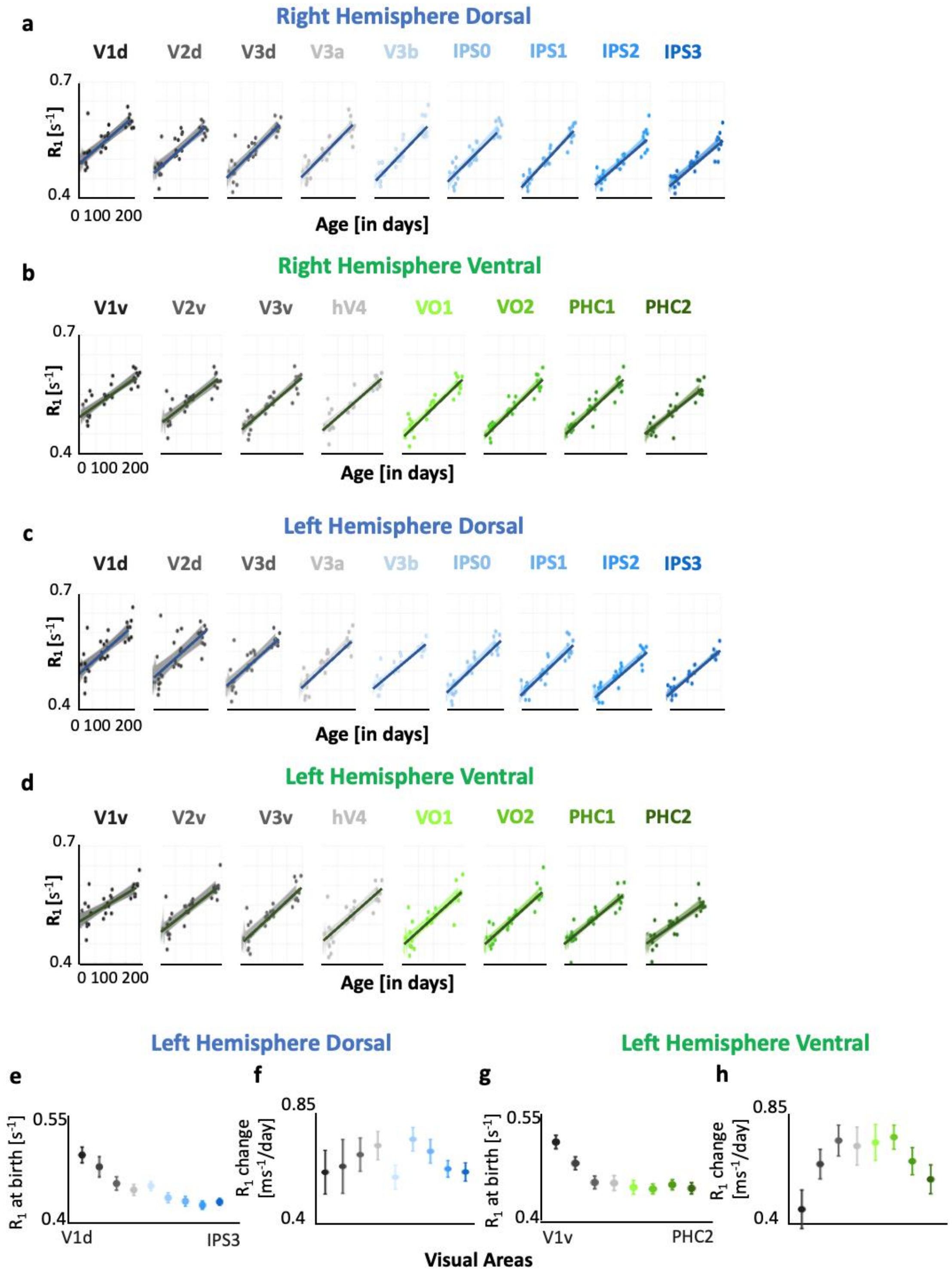

Supplementary Figure 8. Hierarchical development of R_1 in the dorsal and ventral visual streams during early infancy. (a-d). Developmental trajectory of R_1 in the first 6 months of life, in the right dorsal (a), right ventral (b), left dorsal (c), and left ventral (d) visual streams. R_1 linearly increases from 0 to 6 months of age in all visual areas. *Each dot:* mean R_1 per ROI per infant. *Lines:* LMM fits; *Shaded Area:* 95% confidence interval. For figs. a-d, N_{total} = 30, 10 infants at each time point (newborn, ~3-month-old, ~6-month-old). (e,g) estimated R_1 at birth (LMM Intercepts) and (f,h) estimated R_1 rate of change (LMM slopes) in the left hemisphere. Right hemisphere data are in **Fig. 4**. *Error bars:* standard error on estimates of intercepts and slopes. Slopes and p-values in **Supplementary Table 9**.

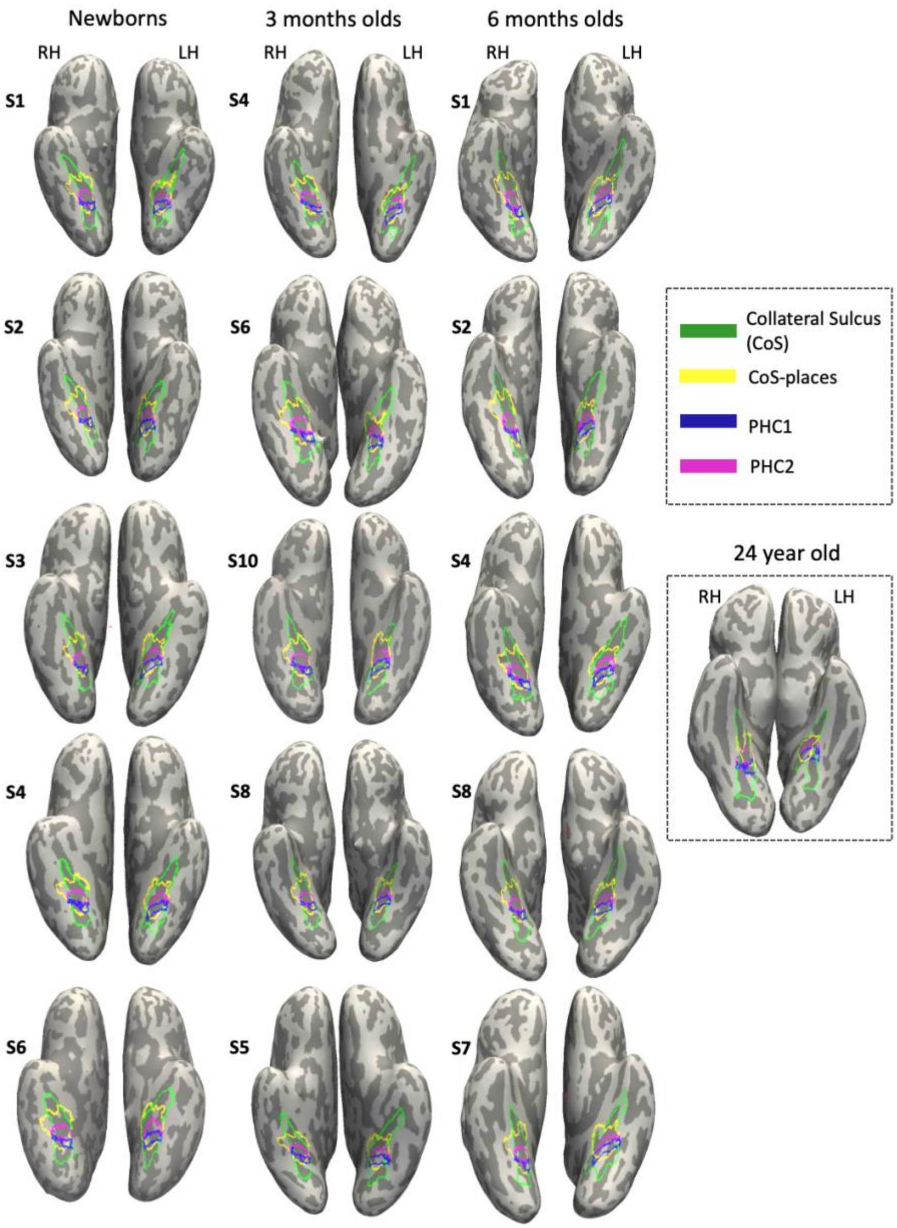

Supplementary Figure 9. Cortex-based alignment of high-level visual regions to individual infants' brains maintain structural-functional and functional-functional relationships. Each panel shows an inflated cortical surface of an individual infant and timepoint on which is projected a cortex-based alignment of Desikian[1] CoS (green), Rosenke[2] CoS-places (yellow), Wang[3] PHC1 (blue), and Wang[3] PHC2 (cyan). There is a consistent overlap between the collateral sulcus (CoS), place-selective region CoS-places, and retinotopic areas PHC1 and PHC2 in infants across time points (newborn, 3-month-old and 6-month-old). This is the same functional-structural organization as in adults (see example adult brain in the inset on the right). Out of the 60 infant hemispheres, we found only a single right hemisphere where the projection of the CoS from the Desikian[1] atlas was less aligned to the infant's CoS (it was shifted more laterally). However, even in that infant's right hemisphere, the functional regions were aligned to the CoS. This indicates there is a consistent anatomical alignment and a stable functional-structural relationship when mapping from adult atlases to infant brains using cortex-based alignment. Subject numbers (S#) are labeled on the left side of each brain and match the subject numbers in **Supplementary Fig. 2**. The same subject may be repeated across different time points. *LH*: left hemisphere. *RH:* right hemisphere.

Supplementary Table 1. Timepoints completed by infants included in the study.
Green: scanned, *white:* missing timepoint.

Participant	Birth sex	0 month	3 months	6 months
S1	Male			
S2	Male			
S3	Female			
S4	Female			
S5	Male			
S6	Female			
S7	Male			
S8	Female			
S9	Male			
S10	Female			
S11	Male			
S12	Female			
S13	Male			

Supplementary Table 2. Statistical significance and parameters of linear mixed models (LMMs) quantifying the relationship between mean T_1 and age [in days] in primary sensory cortices (related to **Fig. 1** and **Supplementary 3**). *LH/RH:* left/right hemisphere; Primary Visual (V1); Primary Auditory (A1); Primary Somatosensory (S1); Primary Motor (M1). *InC*: intercept units: seconds [s]; Slope units: [s/day]. R^2 = proportion of variance explained. All values survive Bonferroni correction at *P*<0.001.

Primary Sensory Regions	Slope (LH)	InC (LH)	P-value (LH)	R^2 (LH)	Slope (RH)	InC (RH)	P-value (RH)	R^2 (RH)
V1	-0.0019	2.01	1.48×10^{-9}	0.73	-0.0020	2.03	2.22×10^{-11}	0.82
A1	-0.0013	1.99	1.33×10^{-7}	0.63	-0.0017	2.04	2.63×10^{-9}	0.75
S1	-0.0018	2.09	2.44×10^{-12}	0.89	-0.0020	2.11	9.67×10^{-12}	0.86
M1	-0.0019	2.08	8.86×10^{-13}	0.84	-0.0019	2.10	3.73×10^{-10}	0.87

Supplementary Table 3. Statistical significance and parameters of LMMs quantifying the relationship between mean MD and age [in days] in primary sensory-motor cortices (related to **Supplementary Fig. 4**). *LH/RH*: left/right hemisphere; Primary Visual (V1); Primary Auditory (A1); Primary Somatosensory (S1); Primary Motor (M1). *InC:* intercept, units: [mm^2 s^{-1}]; Slope units: [mm^2 s^{-1}/day]. R^2 = proportion of variance explained. All values survive Bonferroni correction at P<0.001, except those in gray.

Primary Sensory Regions	Slope (LH)	InC (LH)	P-value (LH)	R^2 (LH)	Slope (RH)	InC (RH)	P-value (RH)	R^2 (RH)
V1	$-1.01x10^{-6}$	0.001210	$6.13x10^{-8}$	0.82	$-1.08x10^{-6}$	0.001212	$9.86x10^{-10}$	0.85
A1	$-5.70x10^{-7}$	0.001208	0.01	0.38	$-7.09x10^{-7}$	0.001214	0.002	0.56
S1	$-6.40x10^{-7}$	0.001221	0.001	0.56	$-5.48x10^{-7}$	0.001200	0.0007	0.80
M1	$-9.36x10^{-7}$	0.001280	$8.26x10^{-7}$	0.85	$-1.06x10^{-6}$	0.001297	$2.59x10^{-5}$	0.65

Supplementary Table 4. Statistical significance and parameters of LMMs quantifying the relationship between mean T_1 and age [in days] in dorsal and ventral visual areas (related to **Fig. 2** and **Supplementary Fig. 5**). *LH/RH*: left/right hemisphere; *InC:* intercept, units: [s]; Slope units: [s/day]. R^2 = proportion of variance explained. All values survive Bonferroni correction at P<0.001.

Dorsal Regions	Slope (LH)	InC (LH)	P-value (LH)	R^2 (LH)	Slope (RH)	InC (RH)	P-value (RH)	R^2 (RH)
V1d	-0.0020	2.01	$5.70x10^{-7}$	0.61	-0.0020	2.01	$2.80x10^{-8}$	0.77
V2d	-0.0023	2.08	$7.45x10^{-6}$	0.59	-0.0023	2.12	$2.66x10^{-8}$	0.73
V3d	-0.0026	2.17	$1.08x10^{-8}$	0.79	-0.0027	2.18	$1.94x10^{-9}$	0.84
V3a	-0.0027	2.20	$9.26x10^{-11}$	0.82	-0.0027	2.18	$1.00x10^{-10}$	0.88
V3b	-0.0023	2.18	$9.14x10^{-11}$	0.83	-0.0029	2.23	$8.68x10^{-13}$	0.86
IPS0	-0.0029	2.26	$1.95x10^{-11}$	0.87	-0.0029	2.23	$1.31x10^{-11}$	0.83
IPS1	-0.0028	2.27	$7.79x10^{-12}$	0.91	-0.0030	2.29	$3.58x10^{-15}$	0.89
IPS2	-0.0026	2.30	$1.71x10^{-11}$	0.94	-0.0025	2.27	$1.90x10^{-12}$	0.89
IPS3	-0.0025	2.28	$1.68x10^{-14}$	0.90	-0.0025	2.29	$3.34x10^{-11}$	0.87

Ventral Regions	Slope (LH)	InC (LH)	P-value (LH)	R^2 (LH)	Slope (RH)	InC (RH)	P-value (RH)	R^2 (RH)
V1v	-0.0015	1.96	$3.75x10^{-6}$	0.54	-0.0019	2.01	$8.11x10^{-9}$	0.73
V2v	-0.0022	2.07	$1.25x10^{-8}$	0.82	-0.0021	2.07	$8.06x10^{-9}$	0.85
V3v	-0.0026	2.17	$1.53x10^{-9}$	0.83	-0.0024	2.14	$2.02x10^{-11}$	0.80
hV4	-0.0026	2.18	$5.02x10^{-8}$	0.70	-0.0024	2.15	$2.86x10^{-10}$	0.76
VO1	-0.0027	2.20	$4.81x10^{-9}$	0.80	-0.0027	2.21	$2.28x10^{-12}$	0.83
VO2	-0.0027	2.21	$4.09x10^{-13}$	0.93	-0.0028	2.22	$8.50x10^{-15}$	0.89
PHC1	-0.0025	2.19	$1.59x10^{-10}$	0.81	-0.0026	2.19	$2.28x10^{-13}$	0.87
PHC2	-0.0023	2.22	$1.49x10^{-8}$	0.80	-0.0025	2.21	$3.62x10^{-10}$	0.84

Supplementary Table 5. Statistical significance and parameters of LMMs quantifying the relationship between mean MD and age [in days] in dorsal and ventral visual areas (related to **Supplementary Fig. 6**). *LH/RH*: left/right hemisphere; *InC*: Intercept, units: [mm^2 s^{-1}]; Slope units: [mm^2 s^{-1}/day]. R^2 = proportion of variance explained. All values survive Bonferroni correction at P<0.001.

Dorsal Regions	Slope (LH)	InC (LH)	P-value (LH)	R^2 (LH)	Slope (RH)	InC (RH)	P-value (RH)	R^2 (RH)
V1d	$-1.20x10^{-6}$	0.001202	$1.90x10^{-6}$	0.82	$-1.17x10^{-6}$	0.001185	$1.01x10^{-8}$	0.82
V2d	$-1.65x10^{-6}$	0.001239	$3.30x10^{-10}$	0.84	$-1.45x10^{-6}$	0.001211	$3.19x10^{-11}$	0.82
V3d	$-1.63x10^{-6}$	0.001253	$1.02x10^{-8}$	0.72	$-1.61x10^{-6}$	0.001239	$8.85x10^{-9}$	0.72
V3a	$-1.55x10^{-6}$	0.001251	$6.94x10^{-10}$	0.77	$-1.60x10^{-6}$	0.001255	$3.41x10^{-11}$	0.82
V3b	$-1.62x10^{-6}$	0.001263	$1.15x10^{-11}$	0.83	$-1.66x10^{-6}$	0.001264	$7.67x10^{-11}$	0.80
IPS0	$-1.52x10^{-6}$	0.001266	$8.84x10^{-14}$	0.92	$-1.63x10^{-6}$	0.001281	$5.00x10^{-11}$	0.82
IPS1	$-1.67x10^{-6}$	0.001299	$1.42x10^{-11}$	0.89	$-1.62x10^{-6}$	0.001290	$7.06x10^{-13}$	0.90
IPS2	$-1.66x10^{-6}$	0.001334	$2.88x10^{-9}$	0.82	$-1.70x10^{-6}$	0.001321	$2.35x10^{-10}$	0.80
IPS3	$-1.33x10^{-6}$	0.001303	$3.89x10^{-8}$	0.76	$-1.43x10^{-6}$	0.001301	$1.65x10^{-10}$	0.86

Ventral Regions	Slope (LH)	InC (LH)	P-value (LH)	R^2 (LH)	Slope (RH)	InC (RH)	P-value (RH)	R^2 (RH)
V1v	$-1.08x10^{-6}$	0.001212	$3.40x10^{-7}$	0.78	$-1.14x10^{-6}$	0.001200	$2.92x10^{-7}$	0.68
V2v	$-1.20x10^{-6}$	0.0012648	$6.18x10^{-5}$	0.46	$-9.81x10^{-7}$	0.001239	0.00025	0.57
V3v	$-1.18x10^{-6}$	0.001254	$4.57x10^{-8}$	0.75	$-1.07x10^{-6}$	0.001221	$1.25x10^{-7}$	0.94
hV4	$-1.24x10^{-6}$	0.001247	$7.17x10^{-8}$	0.75	$-1.10x10^{-6}$	0.001206	$2.07x10^{-7}$	0.82
VO1	$-1.23x10^{-6}$	0.001256	$1.47x10^{-9}$	0.89	$-1.33x10^{-6}$	0.001252	$5.91x10^{-10}$	0.92
VO2	$-1.17x10^{-6}$	0.001267	$1.37x10^{-9}$	0.92	$-1.27x10^{-6}$	0.001251	$6.01x10^{-12}$	0.93
PHC1	$-1.06x10^{-6}$	0.001252	$8.55x10^{-8}$	0.90	$-1.29x10^{-6}$	0.001253	$5.13x10^{-11}$	0.88
PHC2	$-1.01x10^{-6}$	0.001264	$4.53x10^{-7}$	0.86	$-1.21x10^{-6}$	0.001264	$5.74x10^{-9}$	0.88

Supplementary Table 6. Demographic information of postmortem human brain tissue samples used for the transcriptomic gene analysis. The data is obtained from the BrainSpan Atlas portal at www.brainspan.org. Developmental stages from mid to late prenatal to early infancy were used for the differential gene analysis. ***[PCW]:*** post conceptual weeks, *[M]:* months (postnatal); Gender, *F:* female. *M:* male; Ethnicity. *E*: European, *A:* African American, *H*: Hispanic.

Subject ID	Age at Death	Gender	Ethnicity
	Prenatal samples [PCW]		
H376.IV.53	19	F	H
H376.IV.51	21	F	E
H376.IV.54	21	M	A
H376.IV.50	22	M	E
H376.V.51	25	F	H
H376.V.52	26	F	A
H376.V.53	37	M	E
	Postnatal samples [M]		
H376.VI.50	4	M	E
H376.VI.51	4	M	E
H376.VI.52	4	M	A

Supplementary Table 7. Brain regions used for the transcriptomic gene analysis. Tissue samples from prenatal and postnatal human primary sensory-motor, temporal, and parietal cortices profiled by RNA sequencing. Primary motor cortex (M1, BA4), primary visual cortex (V1, BA17), primary somatosensory cortex (S1, BA1-3), primary auditory temporal cortex (A1, BA41), posterior inferior parietal cortex (IPC, BA40), posterior superior temporal cortex (STC, BA22), inferior temporal cortex (ITC, BA20); *pcw:* post conceptual weeks, *M:* months (postnatal).

Subject ID	Age	Regions
H376.IV.53	19 pcw	M1C-S1C
H376.IV.53	19 pcw	IPC
H376.IV.53	19 pcw	A1C
H376.IV.53	19 pcw	STC
H376.IV.53	19 pcw	V1C
H376.IV.51	21 pcw	ITC
H376.IV.54	21 pcw	M1C
H376.IV.54	21 pcw	S1C
H376.IV.54	21 pcw	IPC
H376.IV.54	21 pcw	STC
H376.IV.54	21 pcw	ITC
H376.IV.54	21 pcw	V1C
H376.IV.50	22 pcw	M1C
H376.IV.50	22 pcw	S1C
H376.IV.50	22 pcw	IPC
H376.IV.50	22 pcw	A1C
H376.IV.50	22 pcw	STC
H376.IV.50	22 pcw	ITC
H376.IV.50	22 pcw	V1C
H376.V.51	25 pcw	A1C
H376.V.52	26 pcw	V1C
H376.V.52	26 pcw	STC
H376.V.53	37 pcw	M1C
H376.V.53	37 pcw	S1C
H376.V.53	37 pcw	IPC
H376.V.53	37 pcw	A1C
H376.V.53	37 pcw	STC
H376.V.53	37 pcw	ITC
H376.V.53	37 pcw	V1C
H376.VI.50	4 M	ITC
H376.VI.50	4 M	STC
H376.VI.50	4 M	V1C
H376.VI.51	4 M	M1C
H376.VI.51	4 M	A1C
H376.VI.51	4 M	STC
H376.VI.51	4 M	ITC
H376.VI.52	4 M	M1C
H376.VI.52	4 M	S1C
H376.VI.52	4 M	IPC
H376.VI.52	4 M	A1C
H376.VI.52	4 M	STC
H376.VI.52	4 M	V1C
H376.VI.52	4 M	ITC

Supplementary Table 8. The Gene Ontology (GO) list related to Fig. 3C. This GO list includes the information on the biological processes related to the 95 most differentially expressed genes, listed by statistical significance of functional enrichment analysis. Complete gene ontology lists without and with background gene sets can be found on GitHub: https://github.com/VPNL/babies_graymatter.

Category	ID	Name	p-value Bonferroni
GO: Molecular Function	GO:0019911	structural constituent of myelin sheath	$2.05X10^{-5}$
GO: Molecular Function	GO:0042165	neurotransmitter binding	$1.25x10^{-3}$
GO: Biological Process	GO:0098916	anterograde trans-synaptic signaling	$3.06x10^{-12}$
GO: Biological Process	GO:0007268	chemical synaptic transmission	$3.06x10^{-12}$
GO: Biological Process	GO:0099537	trans-synaptic signaling	$3.86x10^{-12}$
GO: Biological Process	GO:0099536	synaptic signaling	$5.36x10^{-12}$
GO: Biological Process	GO:0007267	cell-cell signaling	$2.59x10^{-11}$
GO: Biological Process	GO:0050804	modulation of chemical synaptic transmission	$3.97x10^{-7}$
GO: Biological Process	GO:0099177	regulation of trans-synaptic signaling	$4.08x10^{-7}$
GO: Biological Process	GO:0048167	regulation of synaptic plasticity	$4.91x10^{-4}$
GO: Biological Process	GO:0048666	neuron development	$3.56x10^{-5}$
GO: Biological Process	GO:1900449	regulation of glutamate receptor signaling pathway	$3.56x10^{-5}$
GO: Biological Process	GO:0030030	cell projection organization	$1.84x10^{-3}$
GO: Biological Process	GO:0030182	neuron differentiation	$3.06x10^{-3}$
GO: Biological Process	GO:0042552	myelination	$4.44x10^{-3}$
GO: Biological Process	GO:0032291	axon ensheathment in central nervous system	$4.69x10^{-3}$
GO: Biological Process	GO:0022010	central nervous system myelination	$4.69x10^{-3}$
GO: Biological Process	GO:0008366	axon ensheathment	$4.84x10^{-3}$
GO: Biological Process	GO:0007272	ensheathment of neurons	$4.84x10^{-3}$
GO: Cellular Component	GO:0043005	neuron projection	$2.98x10^{-15}$
GO: Cellular Component	GO:0030424	axon	$4.20x10^{-14}$
GO: Cellular Component	GO:0036477	somatodendritic compartment	$3.48x10^{-11}$
GO: Cellular Component	GO:0045202	synapse	$3.96x10^{-9}$
GO: Cellular Component	GO:0043025	neuronal cell body	$6.12x10^{-9}$
GO: Cellular Component	GO:0044297	cell body	$4.24x10^{-8}$
GO: Cellular Component	GO:0098794	postsynapse	$2.67x10^{-6}$
GO: Cellular Component	GO:0044304	main axon	$1.09x10^{-5}$
GO: Cellular Component	GO:0098793	presynapse	$9.14x10^{-5}$
GO: Cellular Component	GO:0097060	synaptic membrane	$2.76x10^{-4}$
GO: Cellular Component	GO:0030425	dendrite	$3.33x10^{-4}$
GO: Cellular Component	GO:0097447	dendritic tree	$3.33x10^{-4}$

Supplementary Table 9. Statistical significance and parameters of LMMs quantifying the relationship between mean R_1 and age [in days] in dorsal and ventral visual areas (related to **Fig. 4** and **Supplementary Fig. 8**). *LH/RH:* left/right hemisphere; *Inc:* Intercept, units: [s^{-1}]. Slope units: [ms^{-1}/day]. R^2 = proportion of variance explained. All values survive Bonferroni correction at P<0.001.

Dorsal Regions	Slope (LH)	Inc (LH)	P-value (LH)	R^2 (LH)	Slope (RH)	Inc (RH)	P-value (RH)	R^2 (RH)
V1d	0.62	0.49	$3.02x10^{-7}$	0.65	0.59	0.49	$3.78x10^{-8}$	0.79
V2d	0.64	0.48	$1.59x10\text{-}^{-5}$	0.61	0.63	0.46	$9.52x10^{-9}$	0.78
V3d	0.69	0.45	$3.34x10^{-9}$	0.84	0.73	0.45	$2.06x10^{-10}$	0.89
V3a	0.72	0.44	$8.07x10^{-12}$	0.87	0.73	0.45	$5.04x10^{-11}$	0.90
V3b	0.60	0.45	$1.43x10^{-11}$	0.86	0.78	0.44	$1.26x10^{-12}$	0.85
IPS0	0.75	0.43	$1.21x10^{-12}$	0.93	0.76	0.44	$1.14x10^{-12}$	0.88
IPS1	0.70	0.43	$5.69x10^{-12}$	0.92	0.77	0.42	$8.66x10^{-16}$	0.90
IPS2	0.63	0.42	$3.26x10^{-12}$	0.95	0.64	0.43	$5.35x10^{-12}$	0.87
IPS3	0.62	0.43	$2.56x10^{-15}$	0.92	0.62	0.43	$2.29x10^{-11}$	0.88

Ventral Regions	Slope (LH)	Inc (LH)	P-value (LH)	R^2 (LH)	Slope (RH)	Inc (RH)	P-value (RH)	R^2 (RH)
V1v	0.46	0.50	$2.24x10^{-6}$	0.58	0.56	0.49	$2.45x10^{-9}$	0.77
V2v	0.63	0.48	$2.72x10^{-9}$	0.88	0.59	0.47	$2.29x10^{-9}$	0.89
V3v	0.72	0.45	$2.52x10^{-10}$	0.89	0.67	0.46	$4.43x10^{-12}$	0.85
hV4	0.70	0.45	$1.46x10^{-8}$	0.81	0.66	0.46	$3.24x10^{-11}$	0.80
VO1	0.71	0.44	$1.46x10^{-9}$	0.83	0.73	0.44	$1.92x10^{-13}$	0.86
VO2	0.73	0.44	$4.09x10^{-13}$	0.93	0.76	0.44	$1.96x10^{-15}$	0.90
PHC1	0.64	0.45	$9.08x10^{-12}$	0.87	0.70	0.45	$1.54x10^{-13}$	0.90
PHC2	0.57	0.44	$4.25x10^{-9}$	0.85	0.65	0.44	$1.17x10^{-11}$	0.85

References

1. Desikan, R. S. *et al.* An automated labeling system for subdividing the human cerebral cortex on MRI scans into gyral based regions of interest. *Neuroimage* **31**, 968–980 (2006).
2. Rosenke, M., van Hoof, R., van den Hurk, J., Grill-Spector, K. & Goebel, R. A Probabilistic Functional Atlas of Human Occipito-Temporal Visual Cortex. *Cereb. Cortex* **31**, 603–619 (2021).
3. Wang, L., Mruczek, R. E. B., Arcaro, M. J. & Kastner, S. Probabilistic Maps of Visual Topography in Human Cortex. *Cereb. Cortex* **25**, 3911–3931 (2015).
