# Supplementary material for: Infants’ cortex undergoes microstructural growth coupled with myelination during development
Source: Commun Biol. 2021 Oct 14;4:1191. doi: 10.1038/s42003-021-02706-w (PMC8516989; doi:10.1038/s42003-021-02706-w)
Supplement: Supplementary file 3 — Description of Additional Supplementary Files [file 42003_2021_2706_MOESM3_ESM.pdf]

Description of Additional Supplementary Files

File name: Supplementary Data 1

Description: Source data for all main and supplementary figures.
