# Supplementary material for: Infants’ cortex undergoes microstructural growth coupled with myelination during development
Source: Commun Biol. 2021 Oct 14;4:1191. doi: 10.1038/s42003-021-02706-w (PMC8516989; doi:10.1038/s42003-021-02706-w)
Supplement: Supplementary file 5 — Reporting Summary [file 42003_2021_2706_MOESM5_ESM.pdf]

nature research

Corresponding author(s): Kalanit Grill-Spector

Last updated by author(s): 09/17/2021

Reporting Summary

Nature Research wishes to improve the reproducibility of the work that we publish. This form provides structure for consistency and transparency in reporting. For further information on Nature Research policies, see our Editorial Policies and the Editorial Policy Checklist.

Statistics

For all statistical analyses, confirm that the following items are present in the figure legend, table legend, main text, or Methods section.

n/a	Confirmed	
☐	☒	The exact sample size (n) for each experimental group/condition, given as a discrete number and unit of measurement
☐	☒	A statement on whether measurements were taken from distinct samples or whether the same sample was measured repeatedly
☐	☒	The statistical test(s) used AND whether they are one- or two-sided<br>*Only common tests should be described solely by name; describe more complex techniques in the Methods section.*
☐	☒	A description of all covariates tested
☐	☒	A description of any assumptions or corrections, such as tests of normality and adjustment for multiple comparisons
☐	☒	A full description of the statistical parameters including central tendency (e.g. means) or other basic estimates (e.g. regression coefficient) AND variation (e.g. standard deviation) or associated estimates of uncertainty (e.g. confidence intervals)
☐	☒	For null hypothesis testing, the test statistic (e.g. F, t, r) with confidence intervals, effect sizes, degrees of freedom and P value noted<br>*Give P values as exact values whenever suitable.*
☒	☐	For Bayesian analysis, information on the choice of priors and Markov chain Monte Carlo settings
☒	☐	For hierarchical and complex designs, identification of the appropriate level for tests and full reporting of outcomes
☒	☐	Estimates of effect sizes (e.g. Cohen's d, Pearson's r), indicating how they were calculated

Our web collection on statistics for biologists contains articles on many of the points above.

Software and code

Policy information about availability of computer code

Data collection	N/A
Data analysis	Data analysis was performed in MATLAB version 2017b (The MathWorks, Inc.).<br>Quantitative whole brain images of each infant and timepoint were processed with the mrQ pipeline (https://github.com/mezera/mrQ).<br>Each participant's individual brain anatomical template and initial segmentation of gray and white matter was generated using infant FreeSurfer's automatic segmentation code developed for infant data (infant-recon-all; https://surfer.nmr.mgh.harvard.edu/fswiki/infantFS).<br>DMRI data were preprocessed using a combination of tools from mrTrix3 (https://github.com/MRtrix3/mrtrix3)40 and mrDiffusion toolbox (http://github.com/vistalab/vistasoft).<br>Custom code used to generate the main figures is available with publication at https://github.com/VPNL/babies_graymatter.

For manuscripts utilizing custom algorithms or software that are central to the research but not yet described in published literature, software must be made available to editors and reviewers. We strongly encourage code deposition in a community repository (e.g. GitHub). See the Nature Research guidelines for submitting code & software for further information.

Data

Policy information about availability of data

All manuscripts must include a data availability statement. This statement should provide the following information, where applicable:

- Accession codes, unique identifiers, or web links for publicly available datasets
- A list of figures that have associated raw data
- A description of any restrictions on data availability

The data required to generate the main figures is available in the Supplementary Data file and on Zenodo: https://doi.org/10.5281/zenodo.5514324

Field-specific reporting

Please select the one below that is the best fit for your research. If you are not sure, read the appropriate sections before making your selection.

☐ Life sciences ☒ Behavioural & social sciences ☐ Ecological, evolutionary & environmental sciences

For a reference copy of the document with all sections, see nature.com/documents/nr-reporting-summary-flat.pdf

Behavioural & social sciences study design

All studies must disclose on these points even when the disclosure is negative.

Study description	Quantitative experimental
Research sample	16 full-term and healthy infants (7 female) were recruited to participate in this study. Three infants provided no usable data because they could not stay asleep once the MRI sequences started. Thus, we report data from 13 infants (6 female) across three timepoints: newborn (N=10; age: 8-37 days), 3 months (N=10; age: 79-106 days), and 6 months (N=10; age: 167-195 days). Two participants were re-invited to complete scans for their 6-months session that could not be completed during the first try. Both rescans were performed within 7 days and participants were still within age range for the 6-month timepoint.<br><br>The participant population was racially and ethnically diverse reflecting the population of the San Francisco Bay Area, including two Hispanic, nine Caucasian, two Asian, three multiracial (2 Asian and Caucasian; 1 Native Hawaiian or Other Pacific Islander) participants. Seven out of these 13 infants participated in MRI in all three timepoints (0, 3, 6 months).<br><br>Due to the Covid-19 pandemic and restricted research guidelines, data acquisition was halted. Consequently, the remaining infants participated in either 1 or 2 sessions.
Sampling strategy	As we perform analyses in individual infants' brain space. we planned to have between 10 and 20 infant participants. This number of participants is similar to sample sizes in our previous pediatric samples (Golarai Nature Neuroscience 2007, Gomez Science 2017, Natu PNAS 2019) Due to the COVID pandemic, and resulting restricted research guidelines, data collection was halted at the current sample of 16 participants.
Data collection	All included participants completed the multiple scanning protocols needed to obtain anatomical MRI (aMRI), quantitative MRI, (qMRI) and diffusion MRI (dMRI). Data were acquired at two identical 3T GE Discovery MR750 Scanners (GE Healthcare) and Nova 32-channel head coils (Nova Medical) located at Stanford University: (i) Center for Cognitive and Neurobiological Imaging (CNI) and (ii) Lucas Imaging Center. As infants have low weight, all imaging was done with first level SAR to ensure their safety. Study protocols for these scans were approved by the Stanford University Internal Review Board on Human Subjects Research.<br><br>Scanning sessions were scheduled in the evenings close in time to the infants' typical bedtime. Each session lasted between 2.5 – 5 hours including time to prepare the infant and waiting time for them to fall asleep. Upon arrival, caregivers provided written, informed consent for themselves and their infant to participate in the study. Before entering the MRI suite, both caregiver and infant were checked to ensure that they were metal-free and caregivers changed the infants into MR safe cotton onesies and footed pants provided by the researchers. The infant was swaddled with a blanket with their hands to their sides to avoid their hands creating a loop. During sessions involving newborn infants, an MR safe plastic immobilizer (MedVac, www.supertechx-ray.com) was used to stabilize the infant and their head position. Once the infant was ready for scanning, the caregiver and infant entered the MR suite. The caregiver was instructed to follow their child's typical sleep routine. As the infant was falling asleep, researchers inserted soft wax earplugs into the infant's ears. Once the infant was asleep, the caregiver was instructed to gently place the infant on a makeshift cradle on the scanner bed, created by weighted bags placed at the edges of the bed to prevent any side-to-side movement. Finally, to lower sound transmission, MRI compatible neonatal Noise Attenuators (https://newborncare.natus.com/products-services/newborn-care-products/nursery-essentials/minimuffs-neonatal-noise-attenuators) were placed on the infant's ears and additional pads were also placed around the infant's head to stabilize head motion.<br><br>An experimenter stayed inside the MR suite with the infant during the entire scan. For additional monitoring of the infant's safety and lack of motion, an infrared camera was affixed to the head coil and positioned for viewing the infant's face in the scanner. The researcher operating the scanner monitored the infant via the camera feed, which allowed for the scan to be stopped immediately if the infant showed signs of waking or distress. This setup also allowed tracking the infant's motion; scans were stopped and repeated if there was excessive head motion. To ensure scan data quality, in addition to real-time monitoring of the infant's motion via an infrared camera, MR brain image quality was also assessed immediately after acquisition of each sequence and repeated if necessary.
Timing	Data collection began in Spring 2019 and was halted in March 2020 due to the Covid-19 pandemic and resulting restricted research guidelines
Data exclusions	Three infants provided no usable data because they could not stay asleep once the MRI sequences started.
Non-participation	No participants dropped out or declined participation
Randomization	n/a

Reporting for specific materials, systems and methods

We require information from authors about some types of materials, experimental systems and methods used in many studies. Here, indicate whether each material, system or method listed is relevant to your study. If you are not sure if a list item applies to your research, read the appropriate section before selecting a response.

Materials & experimental systems

n/a	Involved in the study
☒	☐ Antibodies
☒	☐ Eukaryotic cell lines
☒	☐ Palaeontology and archaeology
☒	☐ Animals and other organisms
☐	☒ Human research participants
☒	☐ Clinical data
☒	☐ Dual use research of concern

Methods

n/a	Involved in the study
☒	☐ ChIP-seq
☒	☐ Flow cytometry
☐	☒ MRI-based neuroimaging

Human research participants

Policy information about studies involving human research participants

Population characteristics	We acquired longitudinal measurements of anatomical MRI, diffusion MRI (dMRI), and quantitative MRI (qMRI) in young infants during natural sleep. Measurements were done at 3 timepoints: newborn (N=10; age: 8-37 days), 3 months (N=10; age: 79-106 days), and 6 months (N=10; age: 167-195 days). We obtained high-quality data (see methods) from 13 individuals, 7 of which completed all 3 time-points. The participant population was racially and ethnically diverse reflecting the population of the San Francisco Bay Area, including two Hispanic, nine Caucasian, two Asian, three multiracial (2 Asian and Caucasian; 1 Native Hawaiian or Other Pacific Islander) participants.
Recruitment	Expectant mothers and their infants were recruited from the San Francisco Bay Area using social media platforms. We performed a two-step screening process for expectant mothers.<br><br>Mothers were screened over the phone for eligibility based on exclusionary criteria designed to recruit a sample of typically developing infants and second, eligible expectant mothers were screened once again after giving birth.<br><br>Exclusionary criteria for expectant mothers were as follows: recreational drug use during pregnancy, alcohol use during pregnancy (more than 3 instances of alcohol consumption per trimester; more than 1 drink per occasion), lifetime diagnosis of autism spectrum disorder or a disorder involving psychosis or mania, taking prescription medications for any of these disorders during pregnancy, insufficient written or spoken English ability to comprehend study instructions, and learning disabilities.<br><br>Exclusionary criteria for infants were pre-term birth (< 37 gestational weeks), low birth weight (< 2.49 kgs), small height (< 45 cms), any congenital, genetic, or neurological disorders, visual problems, complications during birth that involved the infant (e.g., NICU stay), history of head trauma, and contraindications for MRI (e.g., metal implants).
Ethics oversight	Study protocols were approved by the Stanford University Internal Review Board on Human Subjects Research.

Note that full information on the approval of the study protocol must also be provided in the manuscript.

Magnetic resonance imaging

Experimental design

Design type	anatomical data only, data was aquired during natural sleep
Design specifications	n/a
Behavioral performance measures	n/a

Acquisition

Imaging type(s)	anatomical, quantitative, and diffusion MRI
Field strength	3T
Sequence & imaging parameters	Anatomical MRI: T2-weighted images were acquired and used for tissue segmentations. T2-weighed image acquisition parameters: TE=124 ms; TR = 3650ms; echo train length = 120; voxel size = (0.8mm)^3; FOV=20.5cm; Scan time: 4 min and 5 sec.

Quantitative MRI: Spoiled gradient echo images (SPGRs) were used together with the Inversion-recovery EPI (IR-EPI) sequence to estimate T1 relaxation time (=1/R1 (relaxation rate)) at each voxel and to generate whole-brain synthetic T1-weighted images. We acquired 4 SPGRs whole brain images with different flip angles = 4, 10, 15, 20; TE=3ms; TR =14ms; voxel size=1mm^3; number of slices=120; FOV=22.4cm; Scan time: 4 times ~5 minutes scans. We also acquired multiple inversion times (TI) in the IR-EPI using a slice-shuffling technique: 20 TIs with the first TI=50ms and TI interval=150ms as well as a second IR-EPI with reverse phase encoding direction. Other acquisition parameters are: voxel size=(2mm)^3; number of slices=60; FOV=20cm; in-plane/through-plane acceleration=1/3; Scan time=two times 1:45 min.

Diffusion MRI:We obtained dMRI data with the following parameters: multi-shell, #diffusion directions/b-value = 9/0, 30/700, 64/2000; TE = 75.7 ms; TR=2800ms; voxel size = (2mm)^3; number of slices=60; FOV=20cm; in-plane/through plane acceleration = 1/3; Scan time: 5:08 min. We also acquired a short dMRI scan with reverse phase encoding direction and only 6 b=0 images (scan time 0:20 min).

Area of acquisition: Whole Brain

Diffusion MRI ☒ Used ☐ Not used

Parameters: We obtained dMRI data with the following parameters: multi-shell, #diffusion directions/b-value = 9/0, 30/700, 64/2000; TE = 75.7 ms; TR=2800ms; voxel size = (2mm)^3; number of slices=60; FOV=20cm; in-plane/through-plane acceleration = 1/3; Scan time: 5:08 min. We also acquired a short dMRI scan with reverse phase encoding direction and only 6 b=0 images (scan time 0:20 min).

Preprocessing

Preprocessing software: dMRI preprocessing was performed in accordance with recent work from the developing human connectome project, using a combination of tools from MRtrix3 (github.com/MRtrix3/mrtrix3) and mrDiffusion (http://github.com/vistalab/vistasoft). We: (i) denoised the data using a principal component analysis, (ii) used FSL's top-up tool (https://fsl.fmrib.ox.ac.uk/) and one image collected in the opposite phase-encoding direction to correct for susceptibility-induced distortions, (iii) used FSL's eddy to perform eddy current and motion correction, whereby motion correction included outlier slice detection and replacement and (iv) performed bias correction using ANTs.

The preprocessed dMRI images were registered to the whole-brain T2- weighted anatomy using whole-brain rigid-body registration and alignment quality was checked for all images. dMRI quality assurance was also performed. Across all acquisitions, less than 5% ± 0.72% of dMRI images were identified as outliers by FSL's eddy tool. We found no significant effect of age across the outliers (no main effect of age: F(2,26)=1.97, p=0.16, newborn: 1.07+0.88%; 3 months: 0.4+0.40%; 6 months: 0.67+0.85%), suggesting that the developmental data was well controlled across all time-points.

The data analysis pipeline is summarized in Supplementary Fig. 1. In brief, IR-EPI data were used to estimate T1 relaxation time at each voxel. These data were also used together with the SPGRs to generate synthetic T1-weighted whole brain anatomies of each infant at each timepoint. All data from that timepoint were aligned to this anatomical image. T2-weighted images were used for segmentation of gray-white matter to generate cortical surface reconstructions and dMRI data and diffusional kurtosis imaging was used to estimate MD in each voxel. All infant data were kept in native space as all analyses were performed within-subject and within-timepoint.

Normalization: Data were analyzed in native brain space for greater precision

Normalization template: Data were not normalized

Noise and artifact removal: We (i) de-noised the data using a principal component analysis, (ii) used FSL's top-up tool (https://fsl.fmrib.ox.ac.uk/) and one image collected in the opposite phase-encoding direction to correct for susceptibility-induced distortions, (iii) used FSL's eddy tool to perform eddy current and motion correction; motion correction included outlier slice detection and replacement, and (iv) performed bias correction using ANTs.

Volume censoring: n/a

Statistical modeling & inference

Model type and settings: We modeled T1, R1, and MD development using linear mixed models (LMMs).

First, we modeled mean development within each region of interest (ROI) using LMMs with age as predictor and a random intercept (estimated T1 at birth) for each individual. Likelihood testing determined that LMMs using different slopes for each individual do not better explain the data compared to LMMs using a single slope across individuals.

To test if effects varied across the hierarchy of visual processing streams we performed additional LMMs across all areas of each stream with factors of age, area, and hemisphere. We used model comparison (likelihood ratio tests) to compare between LMMs. LMMs using different slopes and intercepts for each area significantly better explained the T1 and R1 data than LMMs using a single slope and different intercepts per area. Likelihood tests comparing models 1 and 2 revealed that the random intercept/random slope model (model 2) fit the data best for T1 and R1 (in all model comparisons the latter model was significantly better than the former, likelihood test, Ps<0.01; degrees of freedom (ventral): 472; degrees of freedom (dorsal): 532). Thus, we report the parameters of LMMs with random intercepts and rand slopes for these metrics. For MD, results revealed that the random intercept/fixed slope model fit the data best in the ventral stream (in model comparisons the latter model was not significantly better than the former (P=0.87, degrees of freedom (ventral): 440) but the random intercept and and slope model fit the data best in the dorsal stream (P = 0.001, degrees of freedom (dorsal): 496). For consistency, we report the parameters of LMMs with random intercepts and random slopes for MD as well.

A final LMM was run for each metric to test for developmental differences across streams using all visual areas of both streams with factors of age, area, and stream.

Effect(s) tested

Effect of age on T1, R1, and MD (i) for each area, (ii) across visual areas of each visual processing stream , and (iii) across all visual areas of both streams (LMMs see above).

Specify type of analysis: ☐ Whole brain ☐ ROI-based ☒ Both

Anatomical location(s)

We present whole brain maps of T1 at each time point and infant in Figure 1 and Supplementary Figure 2.

Quantitative analyses are regions of interest (ROI) based. Here, we examined quantitative changes in T1, R1, and MD in the gray matter of four primary sensory-motor cortices as well as across visual areas of the ventral and dorsal visual processing streams from 0 to 6 months of age.

In order to delineate these regions in infants, we used independent brain atlases that delineate these regions. Each atlas is available on the FreeSurfer adult average brain and was projected to each individual participant's cortical surface at each timepoint using infant FreeSurfer's cortical-based alignment tool.

We used the Glasser Atlas (2016) to delineate the primary visual (V1), primary auditory (A1), primary motor (M1), and primary somatosensory (S1) cortices. We used the Wang Atlas (2015) to delineate 9 regions spanning the dorsal visual stream (V1d, V2d, V3d, V3a, V3b, IPS0, IPS1, IPS2, and IPS3) and 8 regions spanning the ventral visual stream (V1v, V2v, V3v, hV4, VO1, VO2, PHC1, and PHC2) in each infant's cortical surface.

Statistic type for inference
(See Eklund et al. 2016)

voxel-wise

Correction

Bonferroni correlation was used to adjust (make more stringent) the p-value and correct for multiple comparisons.

Models & analysis

n/a	Involved in the study
☒	☐ Functional and/or effective connectivity
☒	☐ Graph analysis
☒	☐ Multivariate modeling or predictive analysis
